# Interdomain Dynamics via Paramagnetic NMR on the Highly Flexible Complex Calmodulin/Munc13-1

**DOI:** 10.1021/jacs.2c06611

**Published:** 2022-09-09

**Authors:** Niels Karschin, Stefan Becker, Christian Griesinger

**Affiliations:** † Max Planck Institute for Multidisciplinary Sciences, Am Fassberg 11, Göttingen, Niedersachsen D-37077, Germany; ‡ Cluster of Excellence “Multiscale Bioimaging: From Molecular Machines to Networks of Excitable Cells” (MBExC), University of Göttingen, Göttingen D-37075, Germany

## Abstract

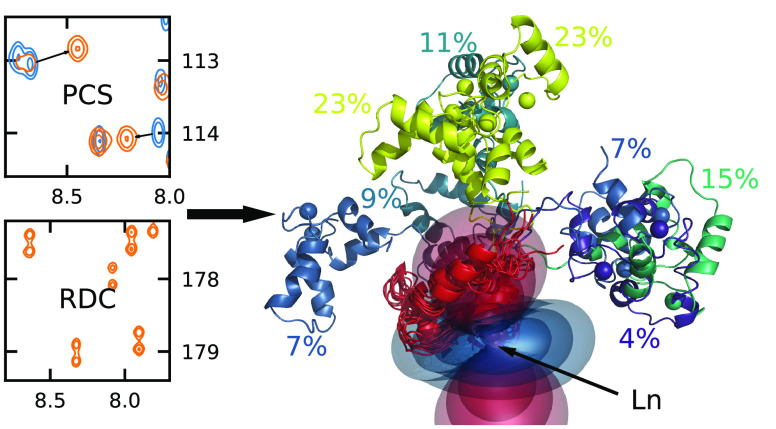

Paramagnetic NMR constraints are very useful to study protein interdomain motion, but their interpretation is not always straightforward. On the example of the particularly flexible complex Calmodulin/Munc13-1, we present a new approach to characterize this motion with pseudocontact shifts and residual dipolar couplings. Using molecular mechanics, we sampled the conformational space of the complex and used a genetic algorithm to find ensembles that are in agreement with the data. We used the Bayesian information criterion to determine the ideal ensemble size. This way, we were able to make an accurate, unambiguous, reproducible model of the interdomain motion of Calmodulin/Munc13-1 without prior knowledge about the domain orientation from crystallography.

## Introduction

The motion of domains within multidomain proteins is often an important component in their function, be it catalysis, channel activation, or molecular recognition,^1−7^ but the study of such motion comes with a number of challenges. This dynamic property should be studied in solution, as crystal packing may strongly distort which part of the conformational space is sampled and highly dynamic proteins can be difficult to crystallize.^8−10^ In cryogenic electron microscopy, flexibility between different domains poses problems as described in a recent review.^11^ Techniques such as Förster resonance energy transfer (FRET) and small-angle X-ray or neutron scattering (SAXS/SANS) can give some insight into this large-scale protein motion, but as they provide only a distribution of a single size parameter (fluorophore distance or radius of gyration, respectively), they are rather low-resolution techniques which miss finer details of the protein motion.^12−15^ Nuclear magnetic resonance (NMR) spectroscopy is a well-established and powerful tool to study various aspects of protein structure and dynamics. However, common constraints such as nuclear Overhauser effects (NOEs), *J* couplings, and chemical shifts are typically poor reporters of interdomain dynamics due to their short range. Examples of effects that provide global orientational constraints in NMR are molecular alignment and anisotropic rotational diffusion, but these come with a severe complication in the presence of interdomain motion.^16−20^ The NMR observables in these cases depend on both the molecular geometry and a tensor (alignment/diffusion tensor), but the large-scale interdomain motion, which changes the overall shape of the protein, has an effect on these tensors. Unfortunately, the contributions to the observables coming from the immediate geometry changes and the indirect effects from the tensor modulation are very difficult to disentangle.

The introduction of a paramagnetic center into the protein induces a number of effects into the NMR spectrum, such as residual dipolar couplings (RDCs) and pseudocontact shifts (PCSs), whose interpretation is conceptually quite similar to alignment or anisotropic diffusion, but come without the unpredictable tensor modulation.^21−25^ The key difference is that this paramagnetic center is located within one of the domains and is therefore rigidly attached to it. In this work, we have the ideal situation that the protein natively contains a binding pocket which can be loaded with paramagnetic lanthanide ions, but there are established methods to label proteins which lack binding pockets.^26−30^ The observed paramagnetic effects within the labeled domain depend on the location of the associated nuclei with respect to this paramagnetic center, but there is (approximately) no relative motion between the two. In the other domains, the paramagnetic effects are modulated by the interdomain motion and one observes the motional average. This averaging reports on the domain motion, and as hundreds of such constraints can potentially be acquired for each domain, this contains a large amount of information about the interdomain dynamics.

The interpretation of this data is not straightforward, as finding a motional model to describe this data is challenging. While internal (intradomain) motion is often described in terms of simple two-state models, this is rarely appropriate for interdomain motion.^31^ Especially in the presence of high degrees of mobility, as is the case with the complex studied in this work, a continuous motional model within the conformational space would seem appropriate. However, available models are restricted, such as uniform motion within a cone, and fail to describe the experimental data.^32^ We therefore fall back to describing the motion as a collection of conformers, i.e., an ensemble.

The various methods for determining ensembles from experimental data can broadly be categorized into two classes.^33,34^ Maximum entropy methods work by perturbing populations of an initial structural ensemble and, loosely speaking, finding the broadest and flattest probability distribution which is in agreement with the data.^35−40^ In contrast, the approach shown in this work is an example of a maximum parsimony method, which follows the principle of Occam’s razor by finding an ensemble with as few members as possible that is in line with experimental constraints. There are various different approaches that have been proposed to determine such ensembles, and they differ in the way the conformations are generated, if and how data uncertainty is considered, in the sampling and selection of ensembles, and how the agreement with the data is balanced with the ensemble size.^41−46^ The approach for sampling the conformational space has to be appropriate for the molecular system and the motion in question, so differences in this aspect arise somewhat naturally. In this context, we introduce a novel way to represent relative domain orientation using homogeneous coordinates. While the concept of scaling data with the associated uncertainty certainly is not new, more often than not this is not done in similar studies, and we advocate it as the only rigorous way to analyze different types of data simultaneously. To find ensembles, we designed a genetic algorithm similar to the work of Nodet et al.,^43^ but in contrast to their work, we incorporated the Bayesian information criterion (BIC)^47^ into the scoring of the ensembles, and as a consequence, the ensemble size emerges naturally from the optimization, which to our knowledge has not been done in this way.

The molecular system we investigated here is a complex of calmodulin with the recognition motif of Munc13-1.^48^ Calmodulin is a calcium-binding, two-domain protein that acts as a calcium sensor and regulates various target proteins depending on the intracellular calcium concentration.^49−57^ The two domains of calmodulin are connected by a flexible linker, and it is well known that they have a high degree of relative mobility in solution.^10,12,13,19,58−62^ Upon complex formation, calmodulin most commonly binds to a short section of an amphiphilic α-helix with both its domains, adopting a collapsed conformation and losing most of its interdomain motion.^63−70^ An example of this is the complex CaM/IQ, which was studied by Russo et al.^25^ also by means of paramagnetic NMR. Munc13-1, in contrast, features a unique 1–5–8–26 binding motif with a long distance between the parts binding to calmodulin’s N- and C-terminal domains, and it is therefore expected that much more of the protein’s domain flexibility is retained.^48,71−74^ Due to this high flexibility it is a challenging problem to characterize this motion, and we show a strategy of how to reproducibly obtain ensemble-like motional models based on paramagnetic NMR constraints.

## Results and Discussion

### Acquisition of Paramagnetic Data

**Figure 1:**
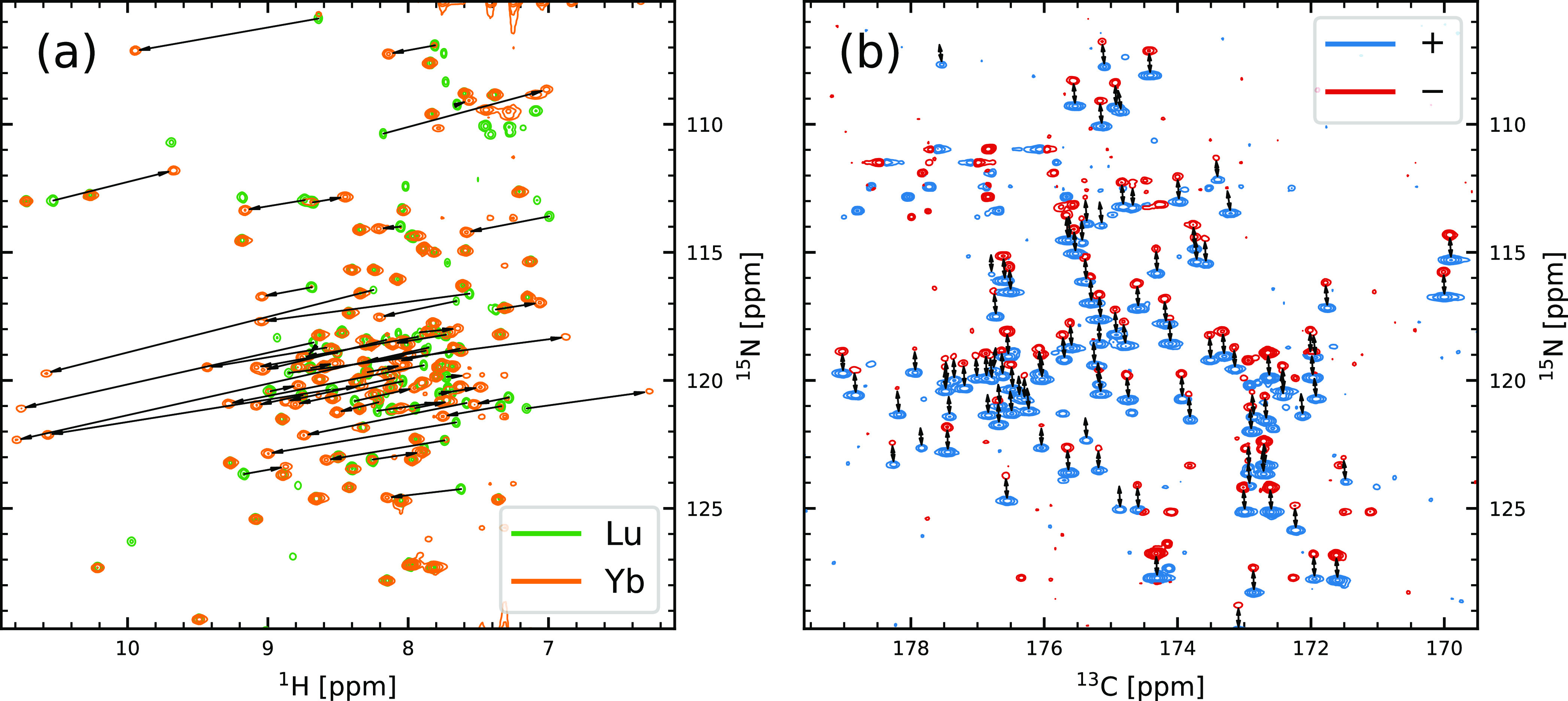
Spectra obtained for the CaM-N60D/Munc13-1 complex loaded with one lanthanide and three Ca^2+^ ions revealing pseudocontact shifts and dipolar couplings: (a) HN projection of HNCO of Lu- and Yb-loaded samples. Arrows emphasize some exemplary PCSs caused by the paramagnetic ion. (b) CN projection of HNCO without H decoupling (Yb). E.COSY-type, antiphase doublets are split by *T*_HC′_ and *T*_HN_ in the carbon and nitrogen dimensions, respectively. Differential relaxation of the ^15^N doublet leads to the fact that some of the negative doublet components are missing at this plot level.

To introduce a lanthanide into calmodulin, we used the well-established N60D mutant, which selectively binds lanthanides in the second binding site within the N-terminal domain.^75^ Neither the mutation nor the lanthanide binding causes significant structural changes, and the complex with Munc13-1 is readily formed.^24,48,75^ Having prepared the complex, it was our goal to acquire as many paramagnetic constraints as possible, within reasonable effort, to maximize the amount of available data. PCSs are determined by simply taking the chemical shift difference between the paramagnetic sample and the diamagnetic reference (Lu), and we determined them for all resonances that were available from the assignment process, namely, amide-H, amide-N, C_α_, and C′ (Figure 1a). RDCs are determined in a similar way. In the spectrum of the diamagnetic reference, only *J* couplings occur, while in the paramagnetic samples, a combined coupling *T* = *J* + *D* is observed. The RDCs are then determined as the difference between the two. We determined all RDCs from 3D NMR spectra, as they reduce peak overlap and have the additional benefit that multiple RDCs can potentially be determined from one experiment (Figure 1b). RDCs in paramagnetic samples are caused by magnetic alignment, so they scale with the square of the magnetic field and therefore benefit from high fields. We acquired the RDCs *D*_HN_ and *D*_HC′_ at 950 MHz and *D*_C′C_α__ and *D*_C_α_H_α__ at 900 MHz.

The amount of paramagnetic constraints that could be extracted from this collection of spectra is summarized in Table 1. It is evident that far fewer data could be collected in the N-terminal domain of calmodulin than in the C-terminal domain, which is due to paramagnetic broadening of the corresponding resonances. Metals with larger susceptibilities (e.g., Dy) provide fewer constraints than lanthanides with a smaller susceptibility (e.g., Yb) for the same reason. This does not make them less valuable, however, since the effects are also larger and therefore less affected by noise, providing better constraints. It is also worth noting that the aim of this work is to analyze the protein’s interdomain motion, and the paramagnetic effects that can report on this motion are the ones located in the C-terminal domain.

**Table 1:** Number of Paramagnetic Constraints in CaM/Munc13-1, Separated by Lanthanide, by Type (PCS/RDC), and by the Protein Domain

		Dy	Er	Ho	Tb	Tm	Yb	sum	
N-terminus	PCS	57	103	117	90	129	199	695	1026
	RDC	13	42	58	18	55	145	331	
C-terminus	PCS	237	233	241	230	238	244	1423	2691
	RDC	213	200	213	209	209	224	1268	

### Fitting of N-Terminal Data

The first step in data analysis is the fitting of the N-terminal data to determine the lanthanide’s susceptibility tensors ***Δχ***. For this we need the expressions for the paramagnetic constraints, which is as follows for PCSs^76,77^
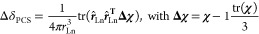
1Here, ***r***_Ln_ is the vector between the lanthanide and the nucleus with PCS and ***r*^**_Ln_ and *r*_Ln_ are the corresponding unit vector and length, respectively. **χ** and ***Δχ*** are the susceptibility tensor and its anisotropic part, respectively. The RDCs obey the following expression^16,77^
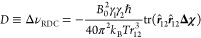
2with the internuclear vector ***r***_12_, the gyromagnetic ratios γ_*i*_, and the temperature *T*. Collecting these equations for each data point gives us a system of linear equations with the five independent components of ***Δχ*** as unknowns, which are chosen as *Δχ*_ax_ = 2*Δχ*_*zz*_ – *Δχ*_*xx*_ – *Δχ*_*yy*_, *Δχ*_rh_ = *Δχ*_*xx*_ – *Δχ*_*yy*_, *Δχ*_*xy*_, *Δχ*_*xz*_, and *Δχ*_*yz*_. The least-squares solution for these components can be found easily and deterministically, and the agreement of the data with the structural model is assessed by a *Q* factor^78^
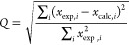
3Here, *x* refers to any type of (weighted) data point (RDC/PCS) and the index indicates whether it is experimental or back-calculated using the fitted susceptibility tensor. We will use subscripts on the *Q* factor to indicate when only a certain type of data was considered (*Q*_PCS_, *Q*_RDC_) and superscripts to differentiate between the two domains (*Q*^N^, *Q*^C^). In this analysis there are still some finer aspects to consider, namely, the choice of the structural model of both the individual domains and the complete peptide/protein complex, the simultaneous evaluation of PCSs and RDCs, the optimization of the lanthanide position, and the inclusion of residual chemical shift anisotropy (RCSA). We will discuss each of these aspects in the following. Whenever we report an improvement in *Q* factor due to any of these points, this was calculated with the other three aspects already optimized.

An accurate structural model is crucial to successfully fit the data and to avoid structural noise.^79^ While there is an NMR structure of the complex CaM/Munc13-1 deposited in the protein data bank (PDB)^80^ with code 2KDU,^48^ it provides a subpar structural model for the N-terminal domain of calmodulin, which is reflected in the comparatively high *Q*^N^ factor (between 0.136 and 0.235) when fitting the data. As there is no crystal structure of CaM/Munc13-1, we chose to use the structure of the complex CaM/IQ with PDB code 2BE6,^81^ which has previously been studied by paramagnetic NMR.^25^ This X-ray ensemble contains three different conformations (A, B, and C), of which 2BE6/B yielded the lowest *Q*^N^ factor against our paramagnetic data (0.0461). We therefore chose to use the N-terminal domain structure of 2BE6/B in all further analyses. Another point concerning the structural model is the fact that small-scale local motion such as bond libration reduces the observed RDC and therefore leads to an underestimation of the tensor size when fitting to a rigid model. A simple way to cope with this is to use larger effective bond lengths. For the most affected RDCs (*D*_NH_ and *D*_C_α_H_α__), we only took the bond orientation from the structure and fixed the length to 1.041 and 1.117 Å, respectively, as described by Ottiger et al.^82^

Equation 3 hides the problem of simultaneously evaluating RDCs and PCSs, which inherently come in different units. A very general way of combining any type of data for simultaneous evaluation is to scale the different data groups with the associated statistical scatter. Although inverse-variance weighting is optimal for normally distributed data, we chose to use the weaker weighting with the inverse standard deviation to account for non-Gaussian behavior. The standard deviation of a group of data was estimated by the root-mean-square deviation (RMSD) against back-calculated data in a fit, and the fits were iteratively repeated with updated weights until convergence (for details, see the SI). These estimated standard deviations were 44 ppb for PCSs and 4.6 Hz for RDCs. Due to the lack of a sufficient number of points in the N-terminal domain, we did not distinguish between different types of RDCs, unlike in the C-terminal domain where more data was available (see below). The uncertainty of PCSs can have a dependence on the lanthanide distance due to paramagnetic broadening as well as structural noise. We investigated this effect and found it to be barely significant (see SI), and we therefore chose not to include it in the weighting of PCSs. The scaled data was then also used for the calculation of *Q* factors (eq 3).

The X-ray structures used for the fit were acquired for a fully calcium-loaded form of wild-type CaM/IQ. For the fit on PCSs, the vector between the lanthanide and the nucleus in question is the relevant geometrical parameter, and in the simplest case, this is determined by simply assuming that the lanthanide takes the exact same position as the calcium ion in the structural model. However, this may not be entirely accurate as the (mutated) binding pocket can adopt a slightly different geometry when binding an ion with different charge and ionic radius. An inaccurate lanthanide position most strongly affects the PCSs from nuclei close to it and results in a poorer fit for these data points. We therefore chose to optimize the lanthanide position with the criterion of minimizing the *Q*_PCS_^N^ factor. This resulted in an improvement in *Q*_PCS_^N^ from 0.0514 to 0.0375 with a change in lanthanide position of 0.60 Å.

Although the chemical shift perturbation caused by the paramagnetic lanthanide is dominated by the PCS, there is a small contribution from alignment in the form of an RCSA as well. Both the PCS and the RCSA depend (apart from the geometry) only on the susceptibility tensor ***Δχ***, and therefore, the inclusion of RCSA comes essentially “for free” in the sense that it does not add additional fit parameters (unlike, e.g., optimizing the lanthanide position). The expression for the change in chemical shift is adapted to^83^
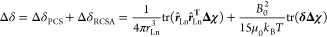
4If this expression is expanded into the aforementioned system of linear equations, the inclusion of RCSAs therefore simply corresponds to a small additional contribution to the linear coefficient matrix. To do this in practice, the chemical shift tensor **δ** is necessary. We chose to include RCSAs only for carbonyl carbon and amide nitrogen nuclei, as these exhibit the largest chemical shift anisotropy. The CSA eigenvalues and the orientation of the eigenvectors within the peptide plane, which we took as the local reference frame for each nucleus, were taken from Loth et al.^84^ This yielded an improvement in the *Q*_PCS_^N^ factor from 0.0467 to 0.0383. It is expected to be even more relevant for the C-terminal domain since the RCSA does not scale with distance like the PCS. Indeed, for the ensembles that we have later found (see below), the RCSA contribution to the change in chemical shift amounts to a remarkable 20% for the C-terminal domain. We will continue referring to the paramagnetically induced chemical shift perturbations as PCSs, but from here on, this will always imply that the RCSA has been included as well.

**Figure 2:**
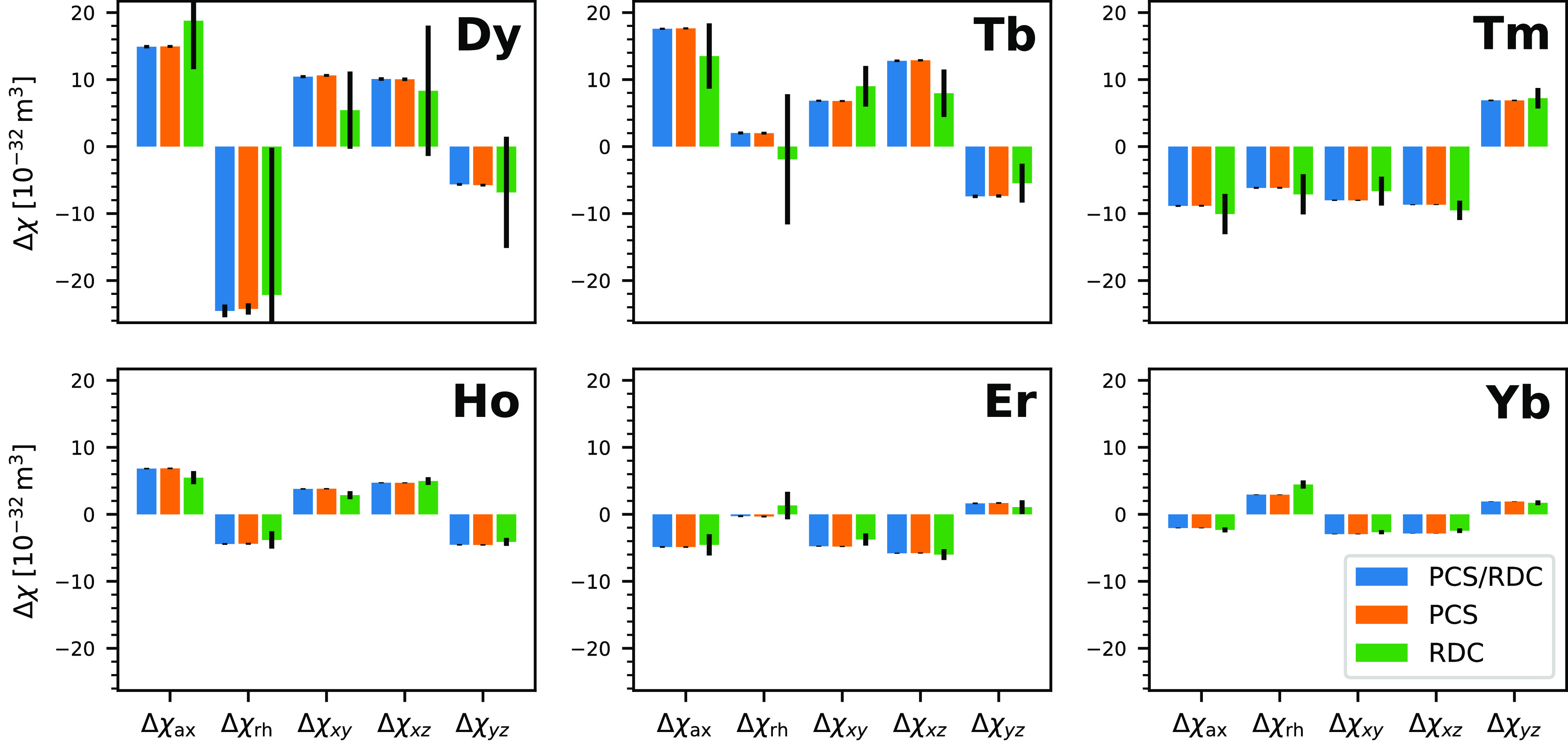
Plot of the five independent tensor elements for the six different lanthanides. Tensor elements obtained from fitting only PCSs or RDCs are displayed as well. Both are in good agreement with each other, except for Dy and Tb, where very few RDCs are available. This leads to a high uncertainty in the fitted tensor elements. The reference coodinate system was taken from the crystal structure 2BE6/B. This data is also tabulated in Table 2.

Taking into account all of these previous considerations, we fitted the N-terminal paramagnetic data to yield the susceptibility tensors ***Δχ*** of the six lanthanide ions. The full tensor has an orientation in space and is therefore dependent on the coordinate system. All structures of CaM/Munc13-1 used in this work were aligned with the backbone of the N-terminal domain to the crystal structure 2BE6/B, which served as a reference frame in this way. The uncertainty of these tensor elements was determined via 1000 steps of bootstrap resampling of the data, a simple but powerful way to estimate the effect of data scatter on the analysis.^85^ Briefly, in a bootstrapping, analysis points from the original data set are drawn with replacement to yield new, synthetic data sets with the same number of points as the original one. These are then subjected to the same fitting as the original data set, and a statistic of the resulting values (such as ***Δχ***) can be done. The uncertainty in the tensor components is calculated as the RMSD over all bootstrap resamples. The five tensor elements are tabulated in Table 2 and plotted in Figure 2, which also shows the tensor if only one type of data (PCS/RDC) is used for fitting. This demonstrates that both types of data do lead to the same result within the margin of error. The tensors determined from RDCs are associated with a higher uncertainty, first because there are fewer data points available (most notably for Dy and Tb) and second because they are associated with a higher relative scatter. The *Q*_PCS_^N^ factor is 0.038, and the *Q*_RDC_^N^ factor is 0.392, which tells us that the RDCs have 10 times higher relative scatter than the PCSs. The overall *Q*^N^ factor is 0.046. Across the various metals the *Q*^N^ factor is relatively consistent, although there are larger variations for the RDC-only fits. All *Q*^N^ factors are tabulated in Table 3.

**Table 2:** Five Independent Components of ***Δχ***, Given in 10^–32^ m^3^, Obtained from Fitting Both RDCs and PCSs to the N-Terminal Domain and from Fitting RDCs to the C-Terminal Domain for the Six Lanthanides^a^

	domain	*Δχ*_ax_	*Δχ*_rh_	*Δχ*_*xy*_	*Δχ*_*xz*_	*Δχ*_*yz*_
Dy	N	14.90 (24)	–24.54 (95)	10.43 (23)	10.08 (26)	–5.66 (19)
	C	–0.14 (28)	–1.74 (42)	–1.95 (24)	3.16 (29)	–0.49 (24)
Er	N	–4.87 (11)	–0.27 (13)	–4.76 (08)	–5.81 (08)	1.64 (12)
	C	0.15 (16)	1.46 (38)	0.05 (14)	–1.00 (17)	0.55 (17)
Ho	N	6.83 (11)	–4.43 (13)	3.80 (09)	4.71 (07)	–4.55 (10)
	C	–0.07 (23)	–1.42 (40)	–0.69 (17)	1.46 (24)	–0.86 (17)
Tb	N	17.58 (15)	2.02 (22)	6.83 (14)	12.79 (18)	–7.43 (24)
	C	0.24 (30)	–2.80 (53)	–0.09 (23)	2.91 (32)	–2.13 (23)
Tm	N	–8.86 (14)	–6.17 (14)	–8.03 (09)	–8.68 (06)	6.89 (10)
	C	0.73 (25)	3.27 (49)	–0.21 (22)	–1.29 (17)	1.69 (18)
Yb	N	–2.04 (05)	2.94 (04)	–2.96 (04)	–2.86 (02)	1.91 (03)
	C	0.09 (17)	0.60 (28)	0.27 (14)	–0.47 (14)	0.20 (16)

a The uncertainty from bootstrap analysis is given in parentheses.

**Table 3:** *Q* Factors for the Fits to Static Structural Models of CaM/Munc13-1

	N-terminal			C-terminal
	PCS/RDC	PCS	RDC	RDC
Dy	0.036	0.027	0.125	0.516
Er	0.052	0.042	0.353	0.805
Ho	0.049	0.042	0.307	0.749
Tb	0.048	0.039	0.220	0.555
Tm	0.047	0.036	0.381	0.613
Yb	0.043	0.038	0.453	0.933
all	0.046	0.038	0.392	0.625

### Fitting of C-Terminal RDCs

**Figure 3:**
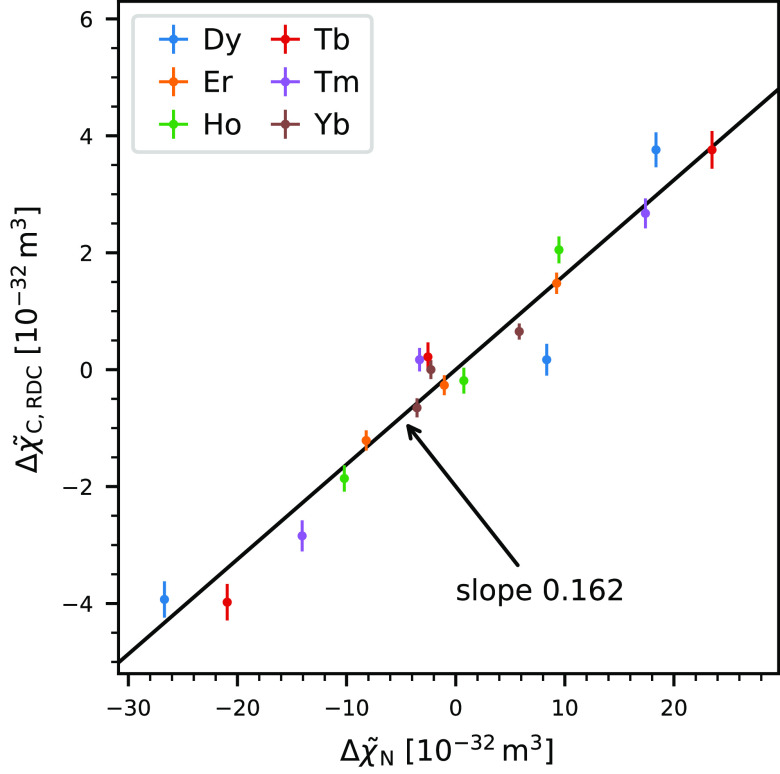
Plot of the eigenvalues of the tensor determined from the N-terminal fit ***Δχ***_N_ against the effective tensor eigenvalues from the RDC-only fit to the C-terminal domain ***Δχ***_C, RDC_. There is a clear linear dependence with a scaling factor of 0.162. Error bars for *Δχ̃*_N_ are too small to be seen.

While it is necessary to have a motional model for the interdomain dynamics to fit PCSs due to their complex, nonlinear dependence on distance to the lanthanide center, this is not true for RDCs. They are an alignment effect, and under the assumption that the C-terminal domain is in itself rigid, one can fit an alignment tensor to the C-terminal RDCs and a domain structure. This alignment tensor can also be expressed as an effective ***Δχ*** tensor, so that it is comparable to the results from the N-terminal domain. Due to the interdomain motion, this C-terminal tensor will be reduced compared to the N-terminal tensor, and this reduction can be used to quantify the motion as a scalar, similar to an order parameter. As a structural model, we used the C-terminal domain of 2BE6/C; although for this analysis the three models of 2BE6 showed very little difference, we later found that 2BE6/C is a significantly better model for the C-terminal domain when including PCSs (see below), so we chose to use this structure here as well. As the RDCs are associated with a relatively high scatter and the size of the RDCs is much smaller than that in the N-terminal domain, these fits produced a very high *Q*_RDC_^C^ factor of overall 0.63 (see Table 3). However, since we acquired a large number of RDCs in the C-terminal domain, the resulting uncertainty in the effective ***Δχ*** tensor was still reasonable. The five tensor components can be found in Table 2. The size reduction of the tensor between the N- and the C-terminal domains can best be seen by plotting their eigenvalues against each other (note that for this correlation the eigenvalues have to be ordered by size and not by absolute value). Figure 3 illustrates that there is a consistent linear scaling between the eigenvalues of the two domain’s tensors with a scaling factor of 0.162 as determined via a least-squares fit. It is not immediately obvious why all metals have a similar scaling, and this is also in contrast to previous findings from Bertini et al., who found 0.15 for thulium and 0.05 for terbium in free calmodulin.^24^ Here, one needs to consider the fact that the anisotropy of the susceptibility tensor is not independent for all metals, as it all originates from the asymmetry of the same binding environment. As a result, the spatial orientation of the ***Δχ*** tensor is similar for all metals, and the mean angle between the eigenframes of our tensors is only around 16°. This does not mean that the tensors themselves are all approximately the same, as the eigenvalues can vary substantially. This is illustrated by looking at the pairwise normalized scalar products of these tensors, which are found in Table S1. Note that the tensors Bertini et al. determined are very similar to those that we found; the normalized scalar products between our and their tensors are 0.98 and 0.97 for Tb and Tm, respectively. The angular difference between the eigenframes of Tm and Tb is as low as 9°, and their normalized scalar product is −0.92, meaning that they are close to being antiparallel. To further investigate this issue, we computationally generated ensembles of three random rotations (the ensemble size that Bertini et al. proposed in their work) and computed the averaged tensors of both Tm and Tb under these three rotations with equal population. We then evaluated individual order parameters for both metals. For only in about 1 in 15 000 of these random rotational ensembles we found the order parameter for Tm to be more than three times bigger than that for Tb. For metal pairs with more different tensors, the distribution is slightly wider (Figure S2), but order parameters varying by a factor of 3 are still hardly encountered. This lets us conclude that it is the norm and not the exception to find order parameters that are approximately equal for all metals. Given that the same metals were used here and by Bertini et al. for the same calmodulin mutant, it is surprising that they observed order parameters varying by such an amount.

### Sampling the Conformational Space of CaM/Munc13-1

A common approach to a motional model of a complex such as Cam/Munc13-1 is to represent it as an ensemble of discrete conformations. The ensemble 2KDU from Rodríguez-Castañeda et al.^48^ was generated without long-distance constraints such as PCSs and RDCs, so it is not surprising that it does not describe the interdomain motion very well and agrees only very poorly with the paramagnetic data we acquired. A simple way to assess the mobility of a given model is to calculate its order parameter, just as we have done in the previous section. This evaluates to be 0.48 for the ensemble 2KDU, which is about a factor of 3 larger than the experimental result, so this ensemble is clearly more rigid than the real complex. To find an ensemble that fits to the experimental data, it was necessary to generate a new pool of conformations from which an ensemble could be compiled. We generated this pool by doing a conformational search with the structures from 2KDU as starting points.

It was our goal to generate a set of conformations that spanned a motional range as large as possible and which only excluded conformations that were sterically impossible to then later choose a subset of this pool as the final model. We therefore did not worry about modeling the protein as realistically as possible or to calculate accurate energies. Instead, we modeled the protein in vacuum using the OPLS3 force field.^86^ We chose this force field since other popular options for proteins, such as AMBER,^87^ did not include parameters for Ca^2+^. The conformational search was executed as a Monte Carlo torsional sampling of the two backbone dihedrals (ϕ, ψ) of the residues 76–81 in calmodulin’s linker region, which corresponds to the region where Tjandra et al. found increased mobility.^19^ Sampling a larger stretch of the linker lead to distortions of the adjacent α-helices, so we limited the search to this relatively small range of residues. The main additional complication compared to a standard conformational search was the presence of the peptide linker, as we needed to ensure that the binding of Munc13-1 was not undone during the search. This was achieved by setting up the sampling such that the N-terminal domain was stationary with respect to Munc13-1 and then adding artificial distance constraints between Munc’s tryptophan 489 and a number of calmodulin’s backbone atoms within the binding pocket. Within the allotted computation time we generated a total of 122 700 conformations of CaM/Munc13-1, which served as a basic pool of conformations in the further analysis. For details concerning the conformational sampling, see the SI.

Such a large number of protein structures can become a challenge to handle computationally, and it was therefore desirable to reduce this data to the essential pieces of information and to discard all others. As explained before, the structure of the individual domains is represented rather poorly by the NMR ensemble 2KDU, and X-ray structures such as 2BE6 agree much better with the available paramagnetic constraints. If we use the domain structures from 2BE6, the only relevant piece of information contained within a conformation of CaM/Munc13-1 is the relative position of the two domains, which can be expressed as three translational and three rotational degrees of freedom. This is obviously an enormous reduction in complexity and facilitates data handling significantly.

This was implemented using homogeneous coordinates, which are a concept from projective geometry that allows us to express both translation and rotation as 4 × 4 matrix multiplication. Using this formalism, we expressed each conformation as the matrix which transforms the C-terminal domain of some reference structure (in our case, 2BE6/B) to the desired location. As an additional feature, it is possible to construct translation-invariant vectors in homogeneous coordinates. This is useful if one has already computed internuclear vectors for RDCs within the C-terminal domain, which are only affected by domain rotation but not by domain translation.

### Ensemble Sampling

After finding this set of sterically allowed domain arrangements, we needed to find a subset that is in agreement with the experimental data, for which we made use of the already accurately determined susceptibility tensors. The first step was to predict the paramagnetic constraints in the C-terminal domain using the ***Δχ*** tensors from the N-terminal fits, the rigid C-terminal domain structure 2BE6/C, and its arrangement relative to the N-terminal domain (and with that the ***Δχ*** tensor) from the transformation matrices, considering also the contribution from RCSAs as explained before. This yielded an array of 122 700 × 2691 data points, and the problem of finding a matching ensemble was now equivalent to finding a linear combination (with certain constraints) of rows of this array that reproduce the 2691 experimental data points. The coefficients of such a linear combination can then be interpreted as populations of the corresponding conformation. Although this is in principle no different from any other system of linear equations, the sheer size of the coefficient matrix of 122 700 × 2691 makes it impossible to find a solution by applying the usual algorithms. We therefore devised a genetic algorithm for this problem, which is a statistical procedure for finding approximate solutions to optimization problems inspired by evolutionary processes. It has the advantage that its runtime does not depend on the size of the available conformations in the pool, making it unnecessary to downsize the rather large number of structures we found in the conformational search.

In this process, a candidate solution is a subset of the 122 700 conformations that we generated in the previous step, which we shall call an ensemble. To calculate the RMSD of an ensemble model against the experimental data, which will be the main component for rating the candidate solutions (i.e., for the fitness function), we need to assign populations to the ensemble members. Arguably, the simplest way is to populate them all equally by the inverse of the number of conformations. This has the advantage that evaluating the fitness (which is typically the computational bottleneck) is very fast. This allows for sampling many more ensembles in the same amount of computation time than when one needs to fit for each population. Larger populations can in principle be described by choosing the same conformation multiple times. However, this approach comes with a number of drawbacks.

First, there is no clear hierarchy in these models, and it is a mistake to equate the number of ensemble members to a formal number of fit parameters. In hierarchical model systems, adding a degree of freedom (i.e., a fit parameter) can never lead to a decrease in agreement as higher level models include lower level models. With adjustable populations, a model with a smaller ensemble size can simply be reproduced by setting some populations to zero. For the case of equally populated ensembles, however, an increase in ensemble size can very well lead to an overall decrease in agreement and slower convergence as too many members lead to a model that is too mobile, and due to the random nature of the sampling, it is rather unlikely to pick the same conformation multiple times. The aspect of model complexity will be discussed more at a later point. Second, finding ensembles with good agreement with the data is much more unlikely, as one needs to find just the right combination, and therefore, the improvement over multiple generations is very slow. It turns out that this effect by far overcompensates for the faster evaluation of the ensemble’s fitness, and therefore, the overall speed of convergence in terms of raw computation time is slower. Third, we find that setting all populations to an equal value is a somewhat artificial choice as conformations of molecules of all sizes are populated to different extents based on their free energy, and we believe it is more realistic to allow for different populations.

We find the populations as a least-squares solution against the experimental data under the constraint that the sum of populations *p*_*i*_ equals one and that there may be no negative populations. The first constraint can be incorporated by adding a row to the coefficient matrix and the data vector with a very high and equal value (see eq S4). The second constraint leads to the non-negative least-squares (NNLS) problem, which can be solved with deterministic algorithms,^88^ and fast implementations are available.^89^

As before with the N-terminal domain, we were again faced with the issue of choosing a structural model and the simultaneous evaluation of PCSs and RDCs. To evaluate PCSs and RDCs simultaneously, we scaled them again with their inverse (estimated) standard deviation. For the RDCs, this was simple as we could use the RMSD from the RDC-only C-terminal fit as an estimate for their scatter. Since there was a significant dependence on the type of RDC, we chose to scale them independently, and their RMSDs were 1.63, 3.42, 2.20, and 0.78 Hz for *D*_NH_, *D*_C_α_H_α__, *D*_HC′_, and *D*_C′C_α__, respectively. For the N-terminal domain, this distinction between RDC types had not been feasible due to the very low number of RDCs for some metals. We estimated the scatter of the PCSs by a short run of the genetic algorithm using exclusively PCSs. After this sampling the RMSD had already converged up to a few percent to the asymptotical value, and we took the resulting RMSD of 9.8 ppb as a reasonable approximation for the PCS scatter. This type of scaling with the estimated standard deviation leads to the effect that the combined, scaled RMSD for a reasonable model will be dimensionless and approach unity, and this is what we will use as the RMSD in the following. We proceeded in a similar way for the three candidates from 2BE6 for the domain structure, and as we found 2BE6/C to give the lowest RMSD, we chose it as our model for the C-terminal domain structure. It is rather surprising to find the PCSs to be the discriminating factor between different domain structures, and it is conventional wisdom that they are less sensitive to small structural variations than RDCs. It is possible that this is simply due to the lower relative uncertainty that is associated with the PCSs. However, as the domain structure was not the scope of this work, we did not investigate this in more detail.

As mentioned before, larger ensembles will always have a lower RMSD, and the discussion so far does not consider the problem of model selection, i.e., determination of an appropriate ensemble size. As this is a crucial part of finding a model, we decided to incorporate this directly into the fitness function using the Bayesian information criterion (BIC).^47^ Under the assumption of independently and normally distributed errors, it can be formulated as^90^
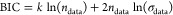
5where *k* is the model’s number of free parameters, *n*_data_ is the number of constraints, and σ_data_ is the data’s standard deviation, estimated by the RMSD. When comparing two models, the one with the lower BIC is preferable. Care has to be taken when counting the number of parameters. If we assume that only the domain structure is a priori knowledge but not the structure of the 122 700 conformations, one needs seven parameters for each ensemble member: six for the degrees of freedom of interdomain orientation and one for the population. The ensemble size *n*_ens_ is fixed to a given number, so it is not immediately obvious how models with different dimensionality should arise. However, it is a feature of the NNLS algorithm that it commonly determines some populations to be exactly zero, and we therefore only consider an effective ensemble size *n*_ens_^eff^ of members with nonzero populations to contribute to the number of parameters. This way, the model dimension *k* = 7*n*_ens_^eff^ is a variable part of the evolving models. To combine the agreement (RMSD) of the model with its dimension, we calculate the fitness *f* as follows
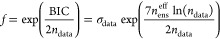
6This fitness is not equal to the BIC, but it yields the same ordering, and it is closer to the more intuitive quantity of an RMSD (σ_data_).

With this fitness function, we can evaluate a set of ensembles and design a genetic process to create subsequent generations. A small fraction of the best ensembles is passed down unchanged to ensure that each generation is at least as good as the previous one. Parents are chosen from the old generation based on an exponential probability. The offspring is then generated by both crossover (combination of two parents) and random mutation (exchange of ensemble members). A more rigorous description can be found in the SI.

**Figure 4:**
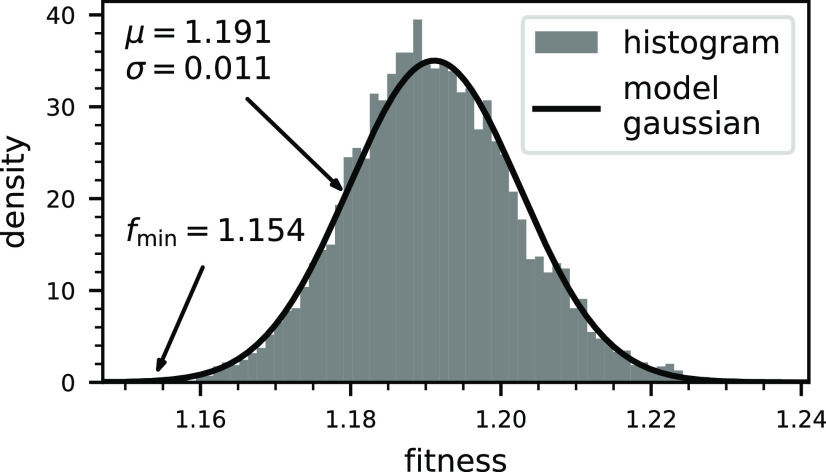
Histogram of the best fitness from the 5994 independent genetic sampling runs. They are, in good approximation, normally distributed with a mean of μ = 1.191 and a standard deviation of σ = 0.011, and the best ensemble found had a fitness of *f*_min_ = 1.154.

This process is then repeated over many generations, mainly limited by computation time. A single run consisted of 1000 generations with a size of 1000 ensembles, which we repeated 5994 times (this seemingly arbitrary number is a consequence of a fixed computing time of 3 days). The ensemble with the best fitness of the last generation is the approximate solution found by a single genetic sampling run. In any type of statistical minimization algorithm it is important to assess the convergence, i.e., how close to a hypothetical true solution we can expect to be. Figure 4 shows a histogram of the fitness of these solutions across all 5994 independent repetitions. This illustrates the distribution of fitness values and lets us estimate how likely it is to find a solution with a given fitness with our algorithm. The next key question in our assessment is what difference in fitness is significant given the imperfect data that we have available, and we have again used bootstrapping to evaluate this. Using 10^6^ bootstrap resamples, we evaluated the statistics of fitness differences between ensembles. They are in very good approximation normally distributed with a standard deviation of 0.0074. Let us now say that we require a 2σ difference in fitness to call two ensembles significantly different, which corresponds to a certainty of around 1 in 20. An ensemble that is significantly better than the best that we have found (*f*_min_ = 1.154) would require a fitness below 1.139. When we compare this to the distribution of results (Figure 4), it is about 4.6σ away from the mean, which corresponds to a frequency of about 1 in 500 000. While it is likely that such a solution exists (there are on the order of 10^56^ possible 11-membered ensembles), it would require 2 orders of magnitude longer sampling and is therefore out of reach of our computational capabilities. We conclude that our sampling has converged to a reasonable degree.

**Figure 5:**
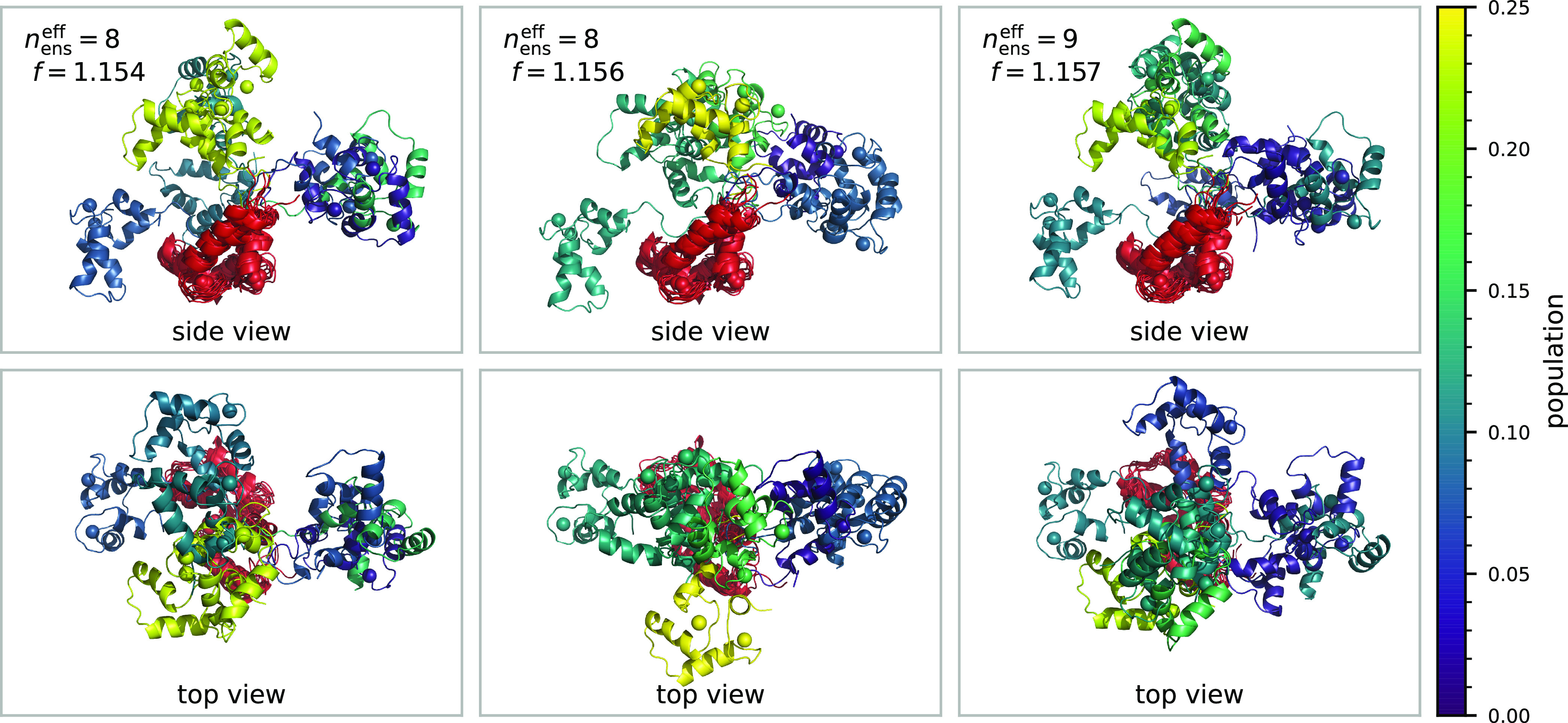
Three best ensembles by fitness found by the genetic algorithm, viewed from two different angles. The N-Terminal domain is depicted in red, while the C-terminus is color coded by population *p*_*i*_.

When comparing the different ensembles found by this procedure, they all populate similar conformations. Figure 5 shows cartoon representations of the three best ensembles by fitness, aligned on the N-terminal domain and shown from two different angles (side and top view). It becomes immediately obvious that the C-terminal domain of CaM/Munc13-1 samples an ample region of space around the N-terminal domain. Seen from the side, this roughly corresponds to an arc of around 180° with the highest population in the center and minor conformations on the sides. When this arc is seen from the top, the appearance is somewhat more distinct, although similarities are still apparent. The best overall ensemble has an effective size of *n*_ens_^eff^ = 8, but within all ensembles that did not significantly differ in fitness we found ensemble sizes between 7 and 11.

**Figure 6:**
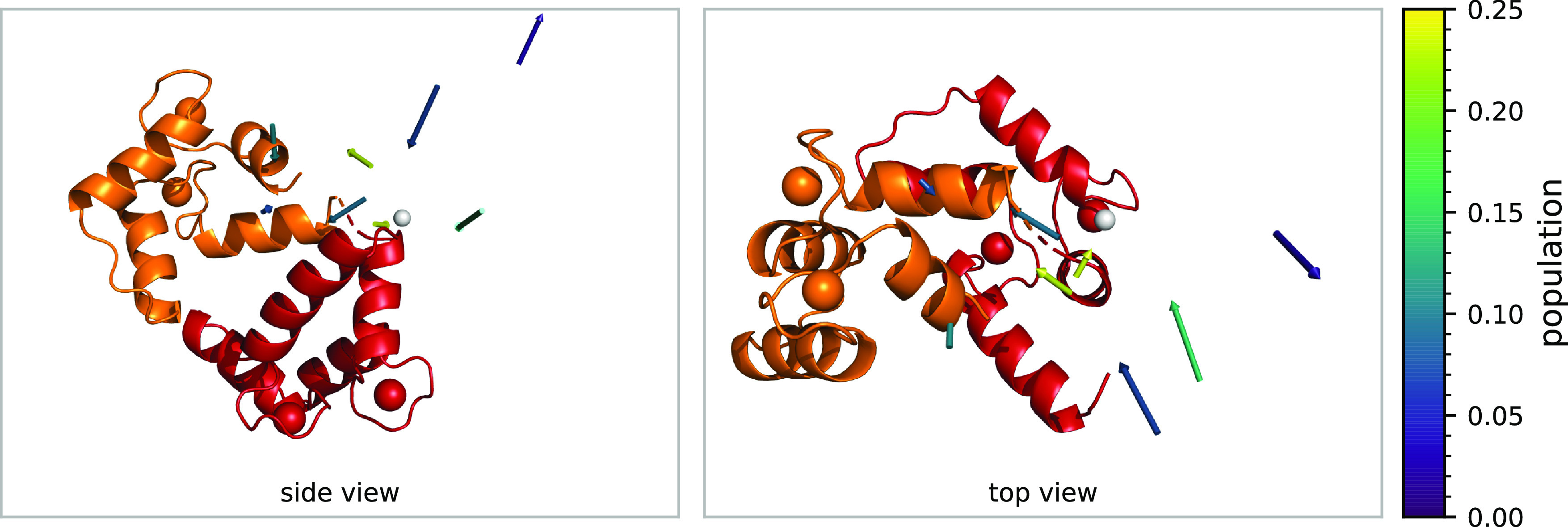
Cartoon of 2BE6/C, and transformations of the best ensemble represented as arrows. Optimal origin of rotation is depicted as a white sphere. The distance between the origin of rotation and the base of each arrow is the remaining translational component, whereas the arrow’s length and orientation represent the angle and axes of rotation, respectively. Arrow color indicates the population *p*_*i*_. This illustrates that no common pivot of rotation can be found.

In all cases, this motion cannot be reduced to rotation along one or two degrees of freedom. In addition to the rotation of one domain around the other, the ensembles show varying degrees of torsion (rotation around a vector connecting the two domains), and none of the ensemble rotations can be reduced to a single pivot. We have shown the latter by trying to shift the origin so that the translational components of an ensemble’s transformation matrices would vanish. However, this proved to be impossible, and the remaining translational components had a mean length of around 13–17 Å, which was quite consistent across the various ensembles. We tried to capture these findings in Figure 6 on the example of the best ensemble (Figure 5, first column). It shows a cartoon of 2BE6/C, which is the starting point for all rotation matrices. The optimal origin of rotation is depicted as a white sphere, which is as expected located in the linker region. We then represented the ensemble members as arrows. The distance between the origin of rotation and the base of these arrows is the remaining translational component, whereas the arrow’s length and orientation represent the angle and axes of rotation, respectively. The population is color coded as before. It is visible from the arrow’s wide distribution how much translation contributes to the domain reorientation. There seems to be no discernible pattern or correlation between translation and rotation, and this image clearly illustrates the wide range of motion that the domains are spanning.

## Summary and Outlook

In this work, we have shown how the interdomain motion of the complex CaM/Munc13-1 could be investigated and modeled using data from paramagnetic NMR. To reduce the impact of unavoidable experimental scatter, we acquired as many paramagnetic constraints in the backbone region as possible and used exclusively triple-resonance experiments to reduce the amount of data rejected because of overlap. This way we have been able to acquire a sizable 1026 constraints in the N-terminus and 2691 constraints in the C-terminal domain. The large amount of data and the careful inclusion of minor effects such as the lanthanide positioning and RCSAs enabled us to determine the lanthanide’s susceptibility tensor from the paramagnetic parameters in the N-terminal domain with a very low relative uncertainty between 1% and 3%, which laid the foundation for subsequent data analysis. Following the simplistic approach from Bertini et al.,^24^ we determined an order parameter of 0.162 by comparing the relative degrees of alignment in both domains. Unlike their results for free calmodulin, we could not find any pronounced dependence on the metal. Due to this inconsistency, it is not clear how the flexibility of CaM/Munc13-1 compares to free calmodulin. When comparing the results to other calmodulin complexes, such as CaM/IQ with an order parameter of around 0.9,^25^ it is obvious that the unique binding motif of Munc13-1 allows for a much wider range of interdomain motion. We also argued that it is expected to find similar order parameters for all metals, since their tensors all share a similar eigenframe (not eigenvalues!). This result should therefore not be interpreted as isotropic motion, which is supported by the fact that the ensembles that we have found to describe our data do not exhibit a high degree of symmetry.

To find these ensembles, we sampled the conformational space of CaM/Munc13-1 extensively using molecular mechanics, including a number of synthetic force contributions to keep the complex together during the sampling. We then devised a way to reduce the information content of each conformation to the relative interdomain arrangement by borrowing a tool from projective geometry, the homogeneous coordinates. From these arrangements and the ***Δχ*** tensors, we predicted the paramagnetic data for each conformation, which yielded the basic data matrix for the subsequent sampling. Compared to the approach of fitting ***Δχ*** tensors against ensembles and C-terminal data, as it was done for CaM/IQ,^25^ this allows the efficient sampling of the large number of conformations that we generated.

This sampling was executed by randomly choosing subsets of conformations and then iteratively improving them using a genetic algorithm, in which we used a fitness function very closely related to the Bayesian information criterion (BIC). This is a computationally fast way to check for overfitting, in contrast to other methods such as cross-validation, and therefore, it could be incorporated directly in the ensemble sampling. In addition, cross validation reduces the number of NMR parameters used to generate the conformational ensemble. As a consequence, we optimized not only conformations and populations but also the appropriate ensemble size, which we found to be around 7–11. Various ensembles with no significant difference in fitness have been found by repeating this nondeterministic process multiple times, and they all span a similar region in space. By examining them, we found that they describe an interdomain motion that comprises both translation on the order of 15 Å and rotation about all three spatial directions. This further confirms our result that the interdomain motion is hardly restricted upon binding to Munc13-1, which is in stark contrast to the case of CaM/IQ which was investigated in a similar manner.^25^ To our knowledge, this is the first time for such a flexible two-domain protein to reproducibly obtain the same ensembles, and we achieved this without prior knowledge about the domain orientation from other techniques such as crystallography.

As we gathered a large amount of paramagnetic constraints of a highly dynamic system, this could be used as a test case for some more fundamental questions about the types of motion that are detectable and distinguishable this way. In the most general way, the interdomain motion can be thought of as a probability density in the six motional degrees of freedom, which is in contrast to the description as an ensemble, which is merely a collection of points in these six dimensions. d’Auvergne et al. already formulated the extensive theory of frame ordering that unifies the description of rotational ordering of rigid body frames, and they found that the averaging of an alignment tensor can be reduced to a rank-4 tensor with 15 independent components, which is therefore the maximum amount of information that can be gathered from RDCs.^32^ PCSs however also encode the distance of the two domains as an inverse third power, so they are both more rich in information and much more difficult to model. d’Auvergne et al. proposed a variety of parametrized, continuous motional models such as the isotropic cone rotation or the free rotor. As none of these models incorporate translation, based on our findings they should not be able to model the motion of CaM/Munc13-1. Also, they do not form clear model hierarchies in the sense that they do not allow incremental increase in model complexity, unlike the ensemble approach, where the ensemble size is such a hierarchical increment. It would be quite interesting to investigate whether it is possible to find a suitable set of 6D functions in which the probability density can be expanded, and whether this approach would be able to outperform an ensemble-based model.

## Materials and Methods

### Sample Preparation

Uniformly ^15^N,^13^C-labeled, protonated N60D-Calmodulin was expressed from *E. coli* following published procedures^91,92^ with the addition of a dialysis step against buffer A (20 mM Bis-Tris, 150 mM KCl, 150 μM CaCl_2_, pH 6.8) to remove remaining ethylenediaminetetraacetate (EDTA). To the resulting solution of 1.8 mL at ∼0.9 mM protein concentration, 50 μL of D_2_O (∼3%) was added for field locking purposes. Ca^2+^ loading was checked via NMR and adjusted by addition of 1 equiv of CaCl_2_ (9.1 μL of 200 mM CaCl_2_). The Munc13-1^458–492^ peptide was prepared by solid-phase synthesis and lyophilized. The complex was prepared by repeatedly adding an aliquot of 500–1000 μL to lyophilized Munc13-1, agitate for 30 min, adjust the pH, and reunite the protein solutions until full 1:1 CaM/Munc13-1-saturation was observed via NMR. This was done to avoid an excess of Munc13-1. The solution was then concentrated to 1120 μL at 1.43 mM with a Vivaspin 20 with a PES membrane and 5 kDa molecular weight cutoff (MWCO). The lanthanide samples were prepared by titrating 30 mM LnCl_3_ in buffer A to 130 μL aliquots of CaM/Munc13-1 until 1:1 Ln loading was observed via NMR with Ln ∈{Lu, Dy, Er, Ho, Tb, Tm, Yb}, with Lu being the diamagnetic reference. The samples were prepared in 3 mm NMR tubes and stored at 4 °C.

### NMR Spectroscopy

All NMR experiments were acquired on Bruker Avance III HD spectrometers operating at proton frequencies between 600 and 950 MHz using inverse cryogenically cooled (QCI/TCI) probes. The sample temperature was set to 298 K and checked using 99.8% MeOD.^93^ Backbone resonance assignment for each sample was done using HNCA^94,95^ spectra using the assignment of the calcium-loaded complex as the starting point.^92^ Triple-resonance experiments were acquired using the following parameters: the carrier offsets were set to 4.7, 116.85, 174.8, and 52.65 ppm and the spectral widths to 14, 28.1, 12, and 28 ppm for H, N, C′, and C_α_, respectively. The number of dummy scans was 512, the number of scans per increment 8, and the relaxation delay 1 s. For the decoupled spectra, the number of acquired real points were 1024, 256, 256, and 128 for H, N, C′, and C_α_, respectively. For the coupled spectra (for RDCs), the numbers of real points were 1024, 320, 352, and 192 for H, N, C′, and C_α_, respectively. All triple-resonance spectra were acquired using nonuniform sampling (NUS),^96^ and the NUS sampling schedule was generated using exponential weighting with effective *T*_2_ times of 50, 50, and 20 ms for N, C′, and C_α_, respectively. For the coupled spectra, a cosine modulation of 52.5 Hz for *T*_C′C_α__ and 143.5 Hz for *T*_C_α_H_α__ was taken into account for the schedule generation. Decoupled HNCO and HNCA spectra were acquired with approximately 10% and 30% sampling density, respectively; coupled HNCO and HNCA spectra for RDCs were acquired with 7% and 15% sampling density, respectively.

^15^N-HSQC spectra were acquired using an in-house sequence with 3–9–19 water suppression,^97^ gradient filters, and ^13^C decoupling during *t*_1_. Decoupled triple-resonance experiments were acquired using standard sequences hncogpwg3d_sct and hncagpwg3d_sct featuring watergate water suppression,^98^ gradient filters, and semiconstant time ^15^N evolution. The coupled experiments were acquired using slight modifications of these sequences. Diagrams of all 3D pulse sequences are found in Figures S4–S6, and all sequences are found in the data collection (SI).

All spectra were processed with NMRpipe^99^ using zero filling to twice the number of points and cosine-squared apodization. NUS reconstruction was done using MddNMR^100^ using the CS-IRLS^101^ algorithm. A sample of processing scripts can be found in the data collection (SI). Peak picking and assignment was done in CCPNMR AnalysisAssign.^102^

### Conformational Search

The conformational search was done in MacroModel^103^ using the 20 structures of 2KDU^48^ as starting points. The search was done as a random sampling of the two backbone angles of residues 76–81. The minimization convergence criterion was 1 kJ mol^–1^ Å^–1^, and the energy cutoff was 3000 kJ mol^–1^. Eighteen backbone atoms within 8 Å of W489-C_δ2_ were constrained using flat-bottom potential wells with 1 Å half-width and a force constant of 200 kJ mol^–1^ Å^–2^. The four hydrogen bonds in the antiparallel β-sheets were constrained using MacroModel’s FXHB operation code. A sample script for the search can be found in the data collection (SI).

### Data Analysis

All computations and data analyses not mentioned elsewhere were performed using a series of self-made python scripts, which make extensive use of the SciPy ecosystem.^104−106^ The paramagnetic data, the transformation matrices for generating the conformers, and the final, cross-validated ensembles can be found in the data collection (SI).

## Supporting Information Available

Additional experimental, methodological, and computational details, including 3D pulse sequences; collection of digital data such as assignments, paramagnetic constraints, ensembles, processing scripts, and pulse sequences (PDF)
