# Supplementary material for: Interdomain Dynamics via Paramagnetic NMR on the Highly Flexible Complex Calmodulin/Munc13-1
Source: J Am Chem Soc. 2022 Sep 9;144(37):17041–53. doi: 10.1021/jacs.2c06611 (PMC9501808; doi:10.1021/jacs.2c06611)
Supplement: Supplementary file 1 — ja2c06611_si_001.pdf [file ja2c06611_si_001.pdf]

Interdomain Dynamics via Paramagnetic NMR on the Highly Flexible Complex Calmodulin/Munc13-1 Supporting Information

Niels Karschin, Stefan Becker, and Christian Griesinger*

Max Planck Institute for Multidisciplinary Sciences, Am Fassberg 11, D-37077 Göttingen, Niedersachsen, Germany

E-mail: cigr@nmr.mpinat.mpg.de

Coupled 3D Spectra

From certain 3D spectra multiple couplings could be extracted. For example, we acquired an HNCO{no H} spectrum without proton decoupling during t_1 and t_2. The resulting doublets are separated in the ^{15}N dimension by T_{HN} and in the ^{13}C dimension by $T_{\mathrm{HC'}}$. The second experiment we acquired was an HNCO{no C_α} without C_α decoupling. This gives $T_{\mathrm{C'C}_\alpha}$ in the carbon dimension and T_{NC_α} in the nitrogen dimension. Since D_{NC_α} is very small due to the relatively large distance and the low gyromagnetic ratios of the nuclei involved, we did not use these couplings as constraints. As a third experiment we acquired an HNCA{no H} without proton decoupling in the t_1 evolution time, which yielded $T_{\mathrm{C}_\alpha\mathrm{H}_\alpha}$ in the carbon dimension. We did not remove proton decoupling during t_2 as this would introduce the unwanted T_{NH} coupling in the nitrogen dimension.

Iterative Weighting

We devised an iterative approach to determine the relative scaling factor of RDCs and PCSs. First, we fitted RDCs and PCSs separately to the structural model, and estimated the associated error by the RMSD of experimental against back-calculated data. We then used these RMSDs as scaling factors to simultaneously fit PCSs and RDCs to the model. From this simultaneous fit, we again determined the RMSDs for PCSs and RDCs, and used them as scaling factors for a new simultaneous fit. This cycle was repeated until the RMSDs did not change any further, which was typically achieved after 3 to 5 iterations. The RMSDs used for scaling converged to 44 ppb for PCSs and 4.6 Hz for RDCs, yielding a relative scaling factor of 105 Hz ppm^{-1}. We did not distinguish between different types of PCSs/RDCs due to the lack of sufficient number of data in the N-terminal domain.

Due to paramagnetic broadening as well as structural noise it is conceivable that there is a dependence of the uncertainty in PCS on the distance to the paramagnetic center. In the case of structural noise small uncertainties in atom position lead to inaccuracies in the back-calculated PCSs, and the magnitude of these inaccuracies will scale with the inverse third power of the distance to the lanthanide. To check for such dependencies we have plotted the difference between experimental and back-calculated PCSs as a function of the lanthanide distance to the third inverse power (Fig. S1a). We have then binned these points in different ranges and calculated the RMS of the deviations as a measure of the PCS uncertainty in this region (Fig. S1b). Although there is a small trend of increasing uncertainty with r_{Ln}^{-3} discernible, we judged it as a minor contribution and therefore chose not to include a distance-dependent weighting factor for the evaluation of PCSs.

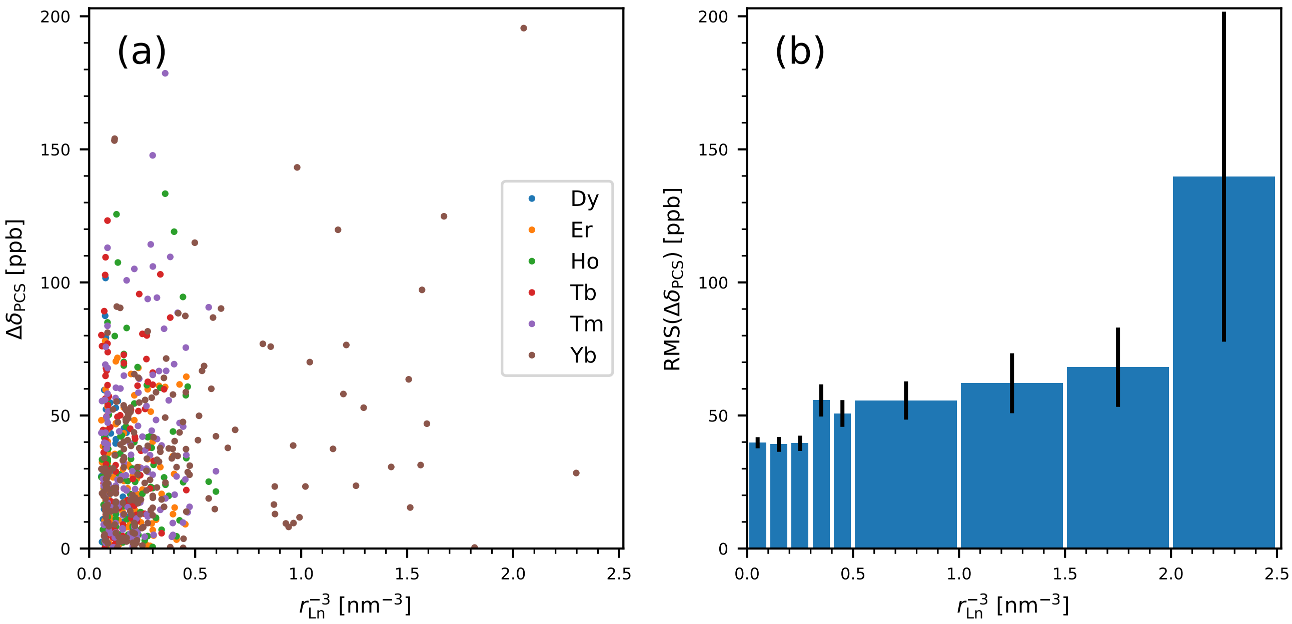

Figure S1: (a) Absolute deviation of experimental and back-calculated PCSs ($\Delta\delta_{PCS}$) against r_{Ln}^{-3}. (b) RMS of $\Delta\delta_{PCS}$ in given ranges of r_{Ln}^{-3}. The step size was changed from $0.1\,nm^3$ to $0.5\,nm^3$ above $0.5\,nm^3$ due to the scarcity of data. Uncertainties were calculated using bootstrapping. Note that the last bin contains only two points, making its value highly uncertain beyond what bootstrapping can capture.

Metal-Specific Tensors and Order Parameters

To compare the similarity of two susceptibility tensors, one can represent these tensors as the vector $\boldsymbol{\chi}$ of their five independent components ($\boldsymbol{\chi} = (\Delta\chi_{ax}\ \Delta\chi_{rh}\ \Delta\chi_{xy}\ \Delta\chi_{xz}\ \Delta\chi_{yz})^{\mathsf{T}}$). Then one computes the normalized scalar products between two such vectors, which are shown in Table S1. To investigate how the different metal tensors scale differently under

Table S1: Matrix of normalized scalar products of the $\boldsymbol{\Delta\chi}$-tensors for the different lanthanides as determined from the fit to the N-terminal domain.

	Dy	Er	Ho	Tb	Tm	Yb
Dy	1.00	−0.62	0.89	0.58	−0.33	−0.92
Er	−0.62	1.00	−0.84	−0.94	0.91	0.82
Ho	0.89	−0.84	1.00	0.86	−0.70	−0.94
Tb	0.58	−0.94	0.86	1.00	−0.92	−0.73
Tm	−0.33	0.91	−0.70	−0.92	1.00	0.61
Yb	−0.92	0.82	−0.94	−0.73	0.61	1.00

dynamic conditions, we generated 1 000 000 ensembles with three uniformly random orientations. We then averaged the different metal tensors using these three orientations and calculated the order parameter S as the scaling factor of the tensor eigenvalues. Then, we calculated pairwise ratios S_1/S_2 for each of the different metals. The distribution of these ratios for all 1 000 000 random ensembles is shown in Fig. S2. As expected, the distribution is narrower for pairs with similar tensors (e.g., Er/Tm), but even for very different tensors it is rare to see order parameter ratios beyond 2 (e.g., 2 % for Dy/Tm). Additionally, we have constructed random ensembles with certain constraints and again looked at the ratio of metal-specific order parameters to see whether higher ratios could be obtained this way. First, we have constrained the three ensemble orientations to be random rotations about one single (random) orientation, i.e. the rotation vector was the same for all three ensemble members (Fig. S3b,e). Second, we have constrained this rotation vector further to be one of the $\boldsymbol{\chi}$-tensors principal axes (Fig. S3c,f). Third, we have constructed six ensembles without any random component as rotations about the principal axes with angles of $\{-30°, 0°, 30°\}$ (Fig. S3c,f, vertical lines). However, all these attempts yielded in more narrow distributions of S-ratios around one compared to the fully random ensembles (Fig. S3a,d). This trend is true both for pairs of tensors which are rather similar (Tm/Tb, Fig. S3a–c) as well as pairs which are rather different (Tm/Dy, Fig. S3d–f).

Conformational Search

The conformational search was executed as a Monte Carlo torsional sampling of certain bond torsion angles. We chose the two backbone dihedrals (ϕ, ψ) of the residues 76 to 81 in calmodulin's linker region to be sampled in the search, which corresponds to the region where Tjandra et al. have found increased mobility.[2] Sampling a larger stretch of the linker lead to distortions of the adjacent α-helices, so we limited the search to this relatively small range of residues. Each sampling step changed a random selection of up to six of the aforementioned torsion angles by up to 10°, followed by a minimization of the resulting structure.

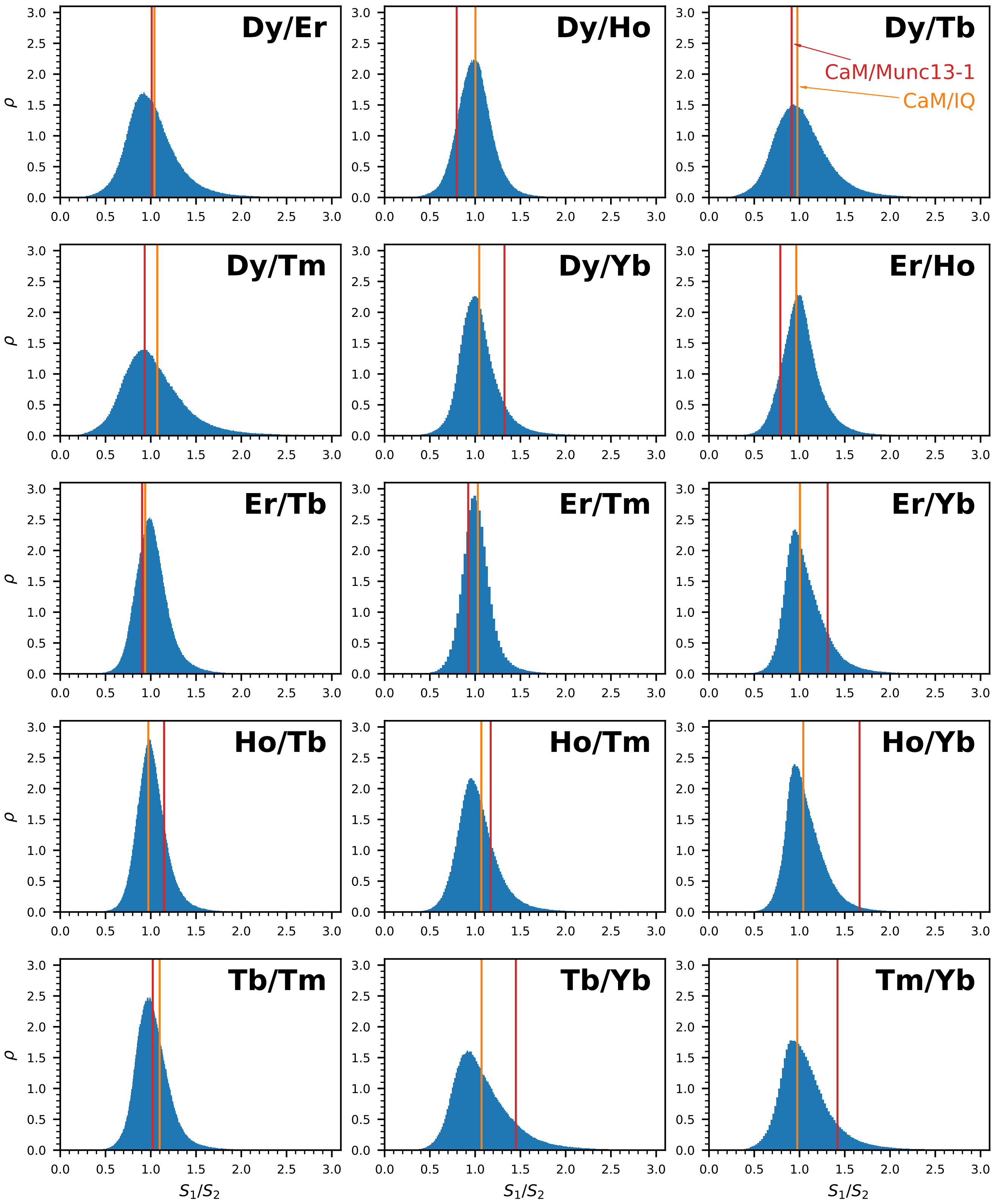

Figure S2: Distribution of order parameter ratios S_1/S_2 in ensembles of three uniformly random orientations for all lanthanide combinations. The vertical lines indicate the experimental values for CaM/Munc13-1 and CaM/IQ.[1]

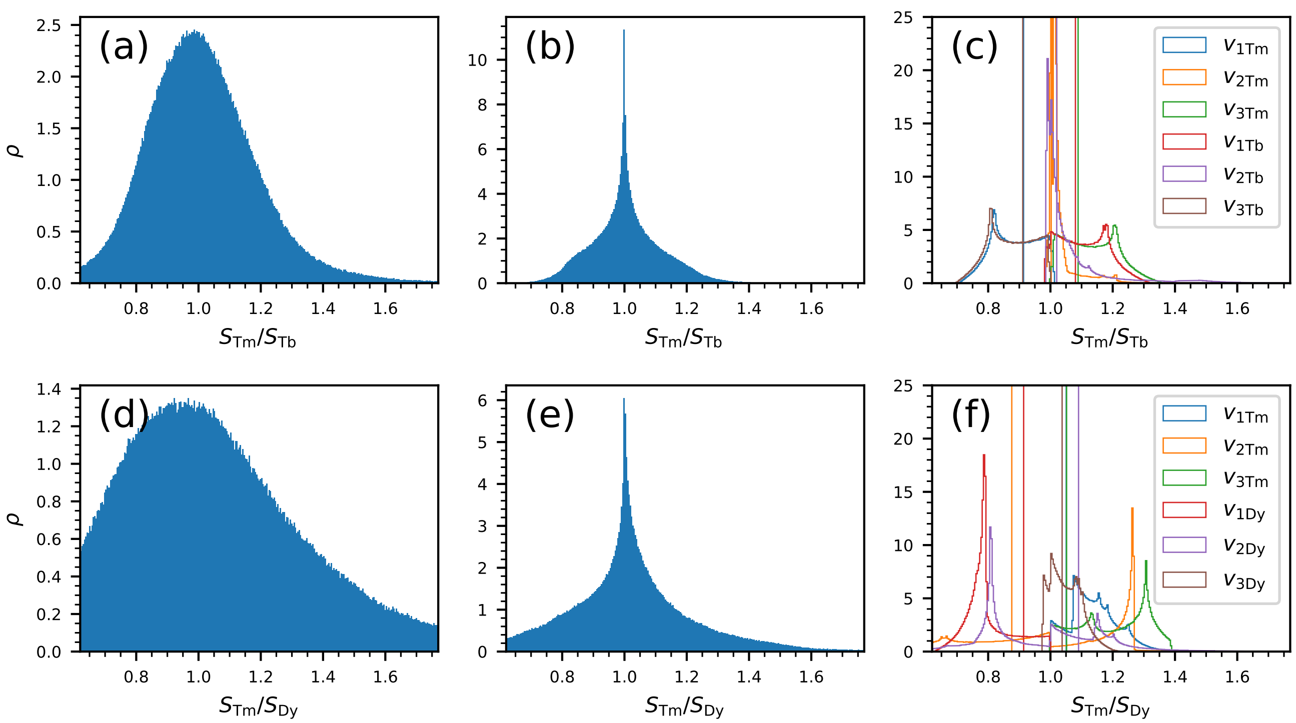

Figure S3: Distribution of order parameter ratios $S_{\mathrm{Tm}}/S_{\mathrm{Tb}}$ in ensembles of (a) three uniformly random orientations, (b) three random rotations about a single random axis, and (c) three random rotations about one of the $\boldsymbol{\chi}$ tensor principal axes. The vertical lines in (c) show the ratio for the set of rotations with angles $\{-30°, 0°, 30°\}$. (d), (e), and (f) are the same plots for the order parameter ratios $S_{\mathrm{Tm}}/S_{\mathrm{Dy}}$.

The convergence criterion was set to a relatively large $1\,\mathrm{kJ\,mol^{-1}\,\AA^{-1}}$ as the minimization was the computational bottleneck of the search. The main additional complication compared to a standard conformational search was the presence of the peptide linker. The change in torsion angles was done such that the N-terminal domain remained immobile, while the C-terminal domain moved through space. We needed to ensure that the binding of Munc13-1 to its pocket in the C-terminal domain was not undone by this step, which was achieved by adding a number of artificial distance constraints. A key residue in this binding is Munc's tryptophan 489, which is located inside of a hydrophobic pocket of calmodulin's C-terminal domain. We added constraints to 18 of calmodulin's backbone atoms that were less than 8 Å away from the central carbon atom $C_{\delta 2}$ of W489. These constraints were executed as flat-bottom potential wells centered around the mean distance of the corresponding atoms in the 20 structures of 2KDU, a half-width of the flat section of 1 Å, and a force constant in the outer part of $200\,\mathrm{kJ\,mol^{-1}\,\AA^{-2}}$. These constraints ensured that after altering the linker torsion angles, W489 was pulled back into its binding pocket in the ensuing minimization step, and distributing this stress onto 18 different atoms prevented a distortion of the binding pocket. Another measure to preserve the original domain structure was constraining the two hydrogen bonds in each domain's section of antiparallel β-sheet. As an energy cutoff we used $3000\,\mathrm{kJ\,mol^{-1}}$, which is twice the difference between the highest and lowest energy structure in the 2KDU ensemble. We ran this conformational sampling with 9900 steps, starting from each of the 20 structures from 2KDU. With approximately two thirds of the steps producing a compatible geometry, we generated a total of 122 700 conformations of CaM/Munc13-1. These served as a basic pool of conformations in the further analysis.

Homogeneous Coordinates

The relative orientation of the two domains and the necessary transformations were implemented using homogeneous coordinates, which are a convenient way to simultaneously

express translation and rotation. For regular three-dimensional coordinates translation is expressed as vector addition and rotation as matrix multiplication. Consider a point P with position vector $\boldsymbol{p}$. Translation and rotations are computed as follows:

$$\boldsymbol{p}_{\mathrm{trans}} = \boldsymbol{p} + \boldsymbol{q}, \qquad \boldsymbol{p}_{\mathrm{rot}} = \mathbf{R}\boldsymbol{p}. \tag{S1}$$

Where $\boldsymbol{q}$ is a translation vector and $\mathbf{R}$ a rotation matrix (i.e., $\mathbf{R}^{\mathsf{T}}\mathbf{R} = \mathbf{1}$ and $|\mathbf{R}| = 1$). However, it is not obvious how to pack these two operations into one, and homogeneous coordinates provide a solution to this problem. They are generated by adding an additional coordinate w, and for any point (x, y, z) the tuple (xw, yw, zw, w) is a set of homogeneous coordinates of this point. Any choice of w refers to the same point, so it is typically set to 1. In these coordinates, both translation and rotation can be expressed as matrix multiplication, as is shown below:

$$\boldsymbol{p}^{\mathrm{homo}}_{\mathrm{trans}} = \begin{pmatrix} 1 & 0 & 0 & q_x \\ 0 & 1 & 0 & q_y \\ 0 & 0 & 1 & q_z \\ 0 & 0 & 0 & 1 \end{pmatrix} \begin{pmatrix} p_x \\ p_y \\ p_z \\ 1 \end{pmatrix} = \begin{pmatrix} q_x + p_x \\ q_y + p_y \\ q_z + p_z \\ 1 \end{pmatrix}, \tag{S2}$$

$$\boldsymbol{p}^{\mathrm{homo}}_{\mathrm{rot}} = \begin{pmatrix} & & & 0 \\ & \mathbf{R} & & 0 \\ & & & 0 \\ 0 & 0 & 0 & 1 \end{pmatrix} \begin{pmatrix} p_x \\ p_y \\ p_z \\ 1 \end{pmatrix} = \begin{pmatrix} \\ \mathbf{R}\boldsymbol{p} \\ \\ 1 \end{pmatrix}. \tag{S3}$$

With this it is possible to compute a matrix representing two or more concatenated translations and rotations by simply taking the matrix product of the individual transformation matrices. Using this formalism we expressed each conformation as a 4×4-matrix, which described the transformation of the C-terminal domain of some reference structure (in our case, 2BE6/B) to the desired location. The transformation matrix was found as the trans-

formation that minimizes the distance RMSD between their backbone atoms (N, C_α, C′) of residues 84 to 145, so both structures were represented as the collection of these atom positions. First, both the reference and the conformation were translated such that the mean of all atom positions would lay in the origin, with the translation matrices $\mathbf{T}_{\text{ref}}$ and $\mathbf{T}_{\text{conf}}$. From there it is a pure rotation $\mathbf{R}$ that transforms the reference into the conformation, which can be found using the Kabsch algorithm.[3] The total transformation matrix $\mathbf{M}$ is then found by concatenating these three operations: $\mathbf{M} = \mathbf{T}_{\text{conf}}^{-1}\mathbf{R}\mathbf{T}_{\text{ref}}$. With these matrices and the knowledge about a single reference structure, any interdomain orientation could be reconstructed. As an additional feature of this formalism, it is possible to construct translation-invariant vectors by setting the coordinate w to zero (i.e., $(x, y, z, 0)^\intercal$). This is useful if one has already computed internuclear vectors for RDCs within the C-terminal domain, which are only affected by domain rotation, but not by domain translation.

Population Fitting: Constraints

The constraint that the sum of populations p_i equals to one can be solved by adding an additional, synthetic data point with a very high value (in our case 10^6) that is the same for both the experimental as well as for all predicted data sets. The system of linear equations then has the following matrix form:

$$\begin{pmatrix} x_{11} & x_{12} & \cdots & x_{1n_{\text{ens}}} \\ x_{21} & x_{22} & \cdots & x_{2n_{\text{ens}}} \\ \vdots & \vdots & \ddots & \vdots \\ x_{n_{\text{data}}1} & x_{n_{\text{data}}2} & \cdots & x_{n_{\text{data}}n_{\text{ens}}} \\ 10^6 & 10^6 & \cdots & 10^6 \end{pmatrix} \begin{pmatrix} p_1 \\ p_2 \\ \vdots \\ p_{n_{\text{ens}}} \end{pmatrix} = \begin{pmatrix} x_{\text{exp},1} \\ x_{\text{exp},1} \\ \vdots \\ x_{\text{exp},n_{\text{data}}} \\ 10^6 \end{pmatrix}. \tag{S4}$$

It is easy to see that the equation for the last row in Eq. (S4) is equivalent to the constraint of $\sum p_i = 1$, and due to the high value small deviations from this lead to a steep increase in the minimization criterion (i.e., RMSD).

Genetic Algorithm: Details

The new generation was constructed from the old population as follows: the best 5 % are transferred to the next generation unaltered. This procedure, commonly referred to as elitism, ensures that the best fitness never worsens over multiple generations. Next, a fraction of 20 % of the old generation is selected at random as possible parents; however, the probability for selection p^i_{parent} depends on the individual's fitness f_i:

$$p^i_{\text{parent}} \propto \exp -\frac{f_i}{f_0} \tag{S5}$$

This ensures that fitter individuals have a higher chances of generating offspring, but it also allows certain poorer individuals to reproduce, which helps to prevent premature convergence on local minima. The parameter $f_0 = 0.03$ is a measure of how strongly the reproduction is dependent on fitness.

The newly generated individuals can be a mixture of two parents, with the crossover probability $p_{\text{co}} = 0.5$, or descend from a single parent. In the former case they will inherit a subset of conformations from one parent and another subset from the other parent, where the relative sizes of these subsets (i.e., the crossover point) is uniformly random. In the case of a single parent all conformations will be inherited from this parent. For each new individual, the parent(s) are chosen randomly and uniformly from the set of all possible parents. In either case these offspring are subjected to mutation, where each conformation has a probability of $p_{\text{mut}} = 0.1$ to be exchanged with an arbitrary conformation from the overall pool at random.

Most of the algorithm's parameters were chosen based on reasonable guesses and not strictly optimized. In trial runs we did not observe a strong dependence of the algorithm's performance on these parameters and therefore we decided not to conduct a more thorough optimization. The parameter f_0 was chosen based on what difference in f is significant (approximately 2σ, details see below). In preliminary runs we determined the optimal effective

ensemble size $n_{\text{ens}}^{\text{eff}}$ to be around 8 to 12, so we set the ensemble size n_{ens}, which acts as an upper limit, to 20. The population size and the number of generations are limited by the computational power, and we set both to 1000, which yielded a runtime in the order of an hour. Finally, we ran 5994 independent repetitions of the genetic sampling (This seemingly arbitrary number is a consequence of a fixed computing time of three days).

Pulse Sequences

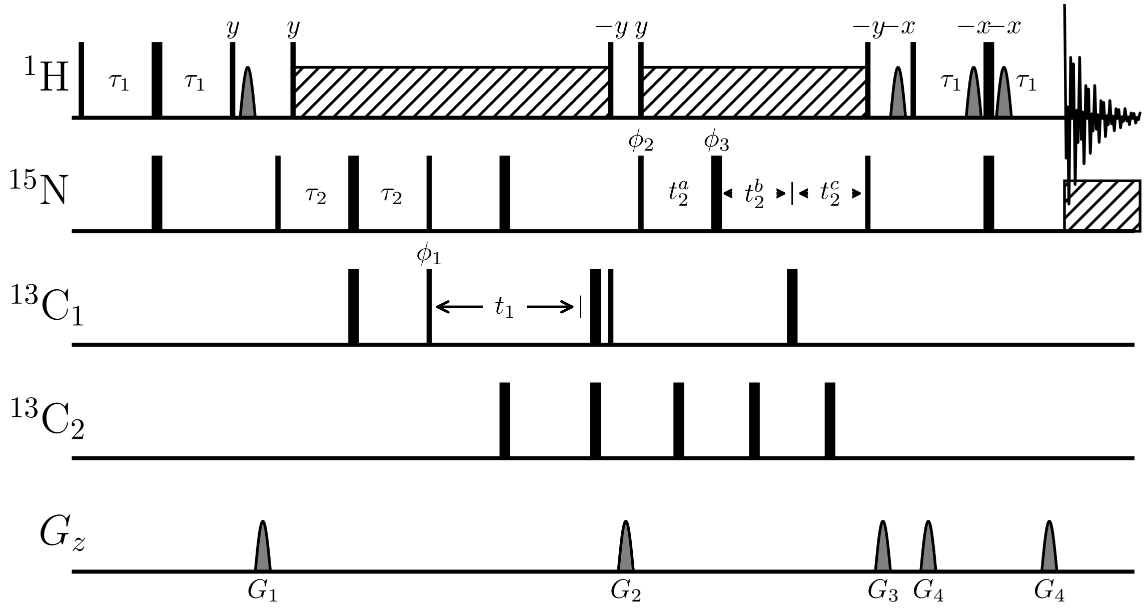

Figure S4: HNCA/HNCO pulse sequence. Cycled phases are $\phi_1 = (x, -x)$, $\phi_2 = (x)_2(-x)_2$, $\phi_3 = (x)_4(-x)_4$, and $\phi_{\text{rec}} = (x, -x, -x, x)_2$. The INEPT delays were set to $\tau_1 = 2.3\,\text{ms}$ and $\tau_2 = 12.4\,\text{ms}$. The semi-constant time delays were $t_2^a = \tau_2 - t_2\tau_2/t_{2,\text{max}}$, $t_2^b = t_2(1/2 - \tau_2/t_{2,\text{max}})$, and $t_2^c = \tau_2 + t_2/2$. For selective C_α and C′ excitation Gauss-cascade type shaped pulses are used,[4,5] and water suppression is achieved via a watergate block in the reverse-INEPT period.[6] For the HNCA, it is $C_1 = C_\alpha$ and $C_2 = C'$, and for the HNCA, it is $C_1 = C'$ and $C_2 = C_\alpha$. The HNCO{no C_α} was acquired by omitting all pulses on C_α.

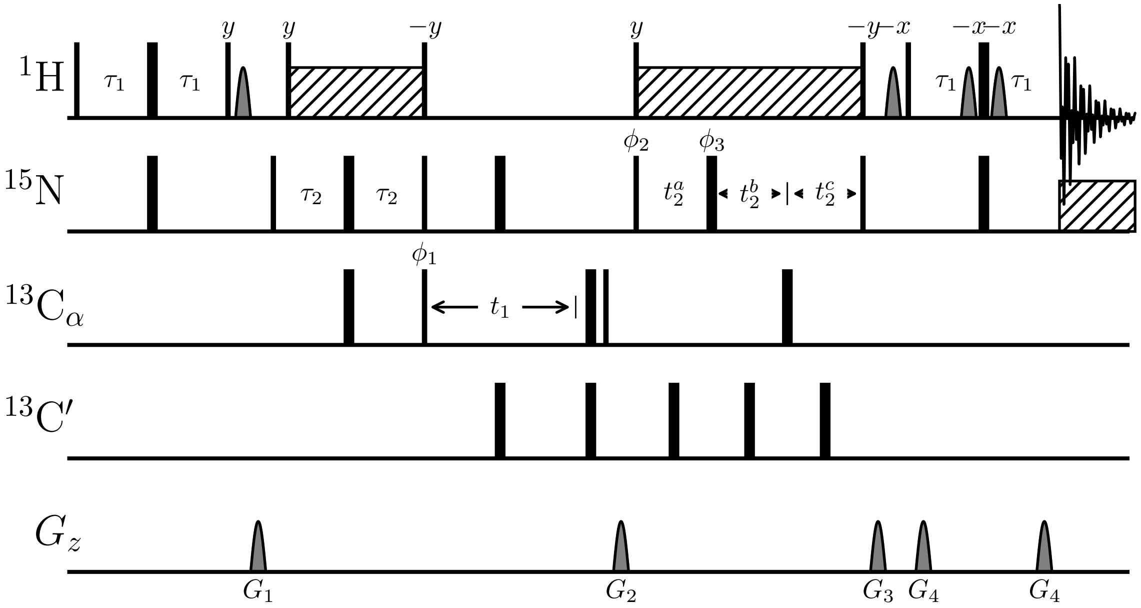

Figure S5: HNCA{no H} pulse sequence. All annotated parameters were identical to the regular HNCA (Fig. S4).

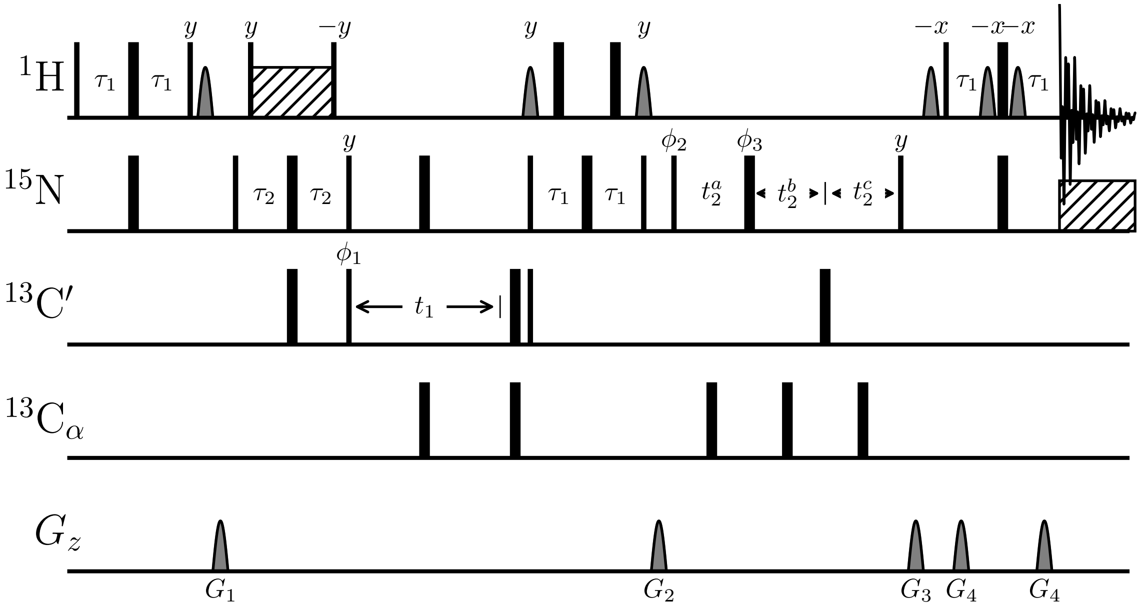

Figure S6: HNCO{no H} pulse sequence. All annotated parameters were identical to the regular HNCO (Fig. S4).

Data Collection

Most numerical and other digital data are too bulky to be printed, and are more conveniently provided as compressed archive of data files. Please click here to open the archive, or access it via the list of attachments of your pdf viewer.

References

(1) Russo, L.; Maestre-Martínez, M.; Wolff, S.; Becker, S.; Griesinger, C. Interdomain Dynamics Explored by Paramagnetic NMR. *J. Am. Chem. Soc.* **2013**, *135*, 17111–17120.

(2) Baber, J. L.; Szabo, A.; Tjandra, N. Analysis of Slow Interdomain Motion of Macromolecules Using NMR Relaxation Data. *J. Am. Chem. Soc.* **2001**, *123*, 3953–3959.

(3) Kabsch, W. A Solution for the Best Rotation to Relate Two Sets of Vectors. *Acta Cryst.* **1976**, *32*, 922–923.

(4) Emsley, L.; Bodenhausen, G. Gaussian Pulse Cascades: New Analytical Functions for Rectangular Selective Inversion and In-Phase Excitation in NMR. *Chem. Phys. Lett.* **1990**, *165*, 469–476.

(5) Emsley, L.; Bodenhausen, G. Optimization of Shaped Selective Pulses for NMR Using a Quaternion Description of Their Overall Propagators. *J. Magn. Reson.* **1992**, *97*, 135–148.

(6) Piotto, M.; Saudek, V.; Sklenář, V. Gradient-Tailored Excitation for Single-Quantum NMR Spectroscopy of Aqueous Solutions. *J. Biomol. NMR* **1992**, *2*, 661–665.
